# Clinical course in corticobasal syndrome and corticobasal degeneration: implications for diagnosis and management

**DOI:** 10.1093/braincomms/fcad321

**Published:** 2023-11-23

**Authors:** Robert I McGeachan, Declan King

**Affiliations:** Centre for Discovery Brain Sciences, The University of Edinburgh, Edinburgh, EH89JZ, UK; UK Dementia Research Institute, The University of Edinburgh, Edinburgh, EH16 4SB, UK; The Hospital for Small Animals, Royal (Dick) School of Veterinary Studies, The University of Edinburgh, Edinburgh, EH259RG, UK; Centre for Discovery Brain Sciences, The University of Edinburgh, Edinburgh, EH89JZ, UK; UK Dementia Research Institute, The University of Edinburgh, Edinburgh, EH16 4SB, UK

## Abstract

This scientific commentary relates to ‘Clinical course of pathologically confirmed corticobasal degeneration and corticobasal syndrome’, by Aiba *et al*. (https://doi.org/10.1093/braincomms/fcad296).

**This scientific commentary relates to ‘Clinical course of pathologically confirmed corticobasal degeneration and corticobasal syndrome’, by Aiba *et al*. (https://doi.org/10.1093/braincomms/fcad296).**

In their recent article in *Brain Communications*, Aiba *et al*.^1^ address the diagnostic challenges associated with corticobasal syndrome (CBS) and corticobasal degeneration (CBD) through an extensive retrospective analysis of a Japanese cohort. Their investigation delves into the clinical presentation and progression of patients who have been confirmed pathologically, genetically, and biochemically to have CBD (*n* = 32). Additionally, the study provides insights into the clinical course of CBS (*n* = 48), with the objective of identifying potential clinical markers indicative of underlying pathology.

## Background

Accurately diagnosing neurodegenerative disorders, including CBD, progressive supranuclear palsy (PSP), and Alzheimer’s disease, remains a formidable challenge. These conditions exhibit a spectrum of clinical presentations with overlapping symptoms, often leading to misdiagnoses. This complicates the ability to predict underlying pathology from clinical presentation, resulting in a low rate of accurate clinical diagnosis^2,3^. For example, CBD can present as progressive supranuclear palsy syndrome (PSPS)^4^ and PSP can present as CBS^5^. This diagnostic uncertainty is not just a matter of semantics; it profoundly affects patients and their families. Accurate prediction of underlying pathology is vital for providing a precise prognosis and is gaining increasing importance as potential therapies emerge in the field of neurodegenerative disorders. Enhancing our capacity to distinguish between the different types of neurodegenerative disorders also plays a pivotal role in optimizing the effectiveness of clinical trials, by ensuring the inclusion of the right patient populations.

## Main findings

Within the research conducted by Abia *et al*., two noteworthy findings emerge in addressing the diagnosis of patients. First, their study confirms that a plethora of neurodegenerative diseases may present with CBS. Corticobasal syndrome had various underlying pathologies, with the most prevalent being CBD at 33.3%, followed by PSP at 29.2%, Alzheimer’s disease at 12.5%, frontotemporal lobar degeneration with TAR DNA-binding protein 43 pathology (FTLD-TDP-43) at 6.3%, globular glial tauopathy at 4.2%, dementia with Lewy bodies at 4.2%, and other conditions at 10.4%. Other diseases included FTLD-fused in sarcoma (FUS), glioblastoma, Pick’s disease, prion disease, and non-specific pathological changes. Therefore, it is crucial from a clinical perspective to recognize that while CBS has historically been closely associated with CBD, only approximately a third of CBS patients in this cohort exhibited pathology characteristics of CBD.

Second, and perhaps more importantly, the study uncovers specific clinical indicators that are valuable for predicting the underlying pathology of CBD, PSP, and Alzheimer’s disease in individuals with CBS. For instance, CBD pathology was accurately predicted with a sensitivity of 81.3% and specificity of 84.4% by the presence of freezing at onset or no dysarthria at presentation and an age at onset of less than 66 years in the case without freezing at onset. PSP pathology was predicted with a sensitivity of 64.3% and specificity of 85.3% when dysarthria at presentation and age at onset older than 61 years were observed. Alzheimer’s disease pathology was predicted with a sensitivity of 66.7% and specificity of 95.2% by pyramidal signs at presentation and personality change during the course of the disease.

## Strengths/limitations

While the reported sensitivity and specificity values in this study are promising, they also underscore the need for caution when interpreting the presence of these clinical signs alone. Given that CBD and PSP are relatively rare diseases when compared to Alzheimer’s disease, these clinical markers may result in a notable number of misdiagnoses if interpreted in isolation. Clinicians should not rely solely on these markers but rather use them as part of their diagnostic toolkit, always considering the individual patient’s case and exercising their clinical judgment. Further, the study focuses on identifying a single pathological background for CBS. However, it is important to note there is a growing body of evidence that suggests that rather than occurring in isolation, combinations of one or more proteinopathies are frequently observed in individuals with neurodegenerative disease and this may complicate diagnosing underlying pathologies^6^. Research has shown that there can be a discrepancy in pathological diagnosis between neuropathologists^7^. Strength of this study is that pathological diagnosis was standardized; all cases were reviewed by a dedicated board of neuropathologists. Sections were assessed for phosphorylated tau (AT8), amyloid-β protein, and with Klüver-Barrera and Gallyas-Braak (G-V) silver staining. As alpha-synuclein and TDP-43 were not stained, it is possible that some co-pathologies were missed.

Some variations are noted, but on the whole, the findings align with various aspects of the Armstrong criteria^8^, reinforcing the utility of the demographic information and clinical signs in predicting CBD. Additionally, the replication of these results in a Japanese cohort is important research and highlights the significance of cross-ethnicity research in establishing the robustness and generalizability of diagnostic criteria.

The quest for earlier and more accurate diagnosis of neurodegenerative disorders remains paramount. Biomarkers are emerging as powerful allies in this pursuit, holding the potential to unveil pathological changes that precede clinical symptoms and differentiate underlying pathologies. However, a limitation of this retrospective study was that it was confined to the information available at the time of data collection, and unable to incorporate all potentially useful biomarkers. Unfortunately, currently available biomarkers for identifying patients with Alzheimer’s disease were not included, which could have influenced the diagnostic accuracy of the clinical markers investigated in this study. The synergistic use of biomarkers and clinical signposts, such as those identified here, presents a promising avenue for enhancing our diagnostic capabilities and bringing us closer to the goal of personalized and targeted medicine.

## Conclusion

The study sheds valuable light on the complex diagnostic landscape of CBS and its relationship to various neurodegenerative pathologies. The diverse range of conditions that may present as CBS emphasizes the critical need for accurate pathology identification. The identified clinical markers, frozen gait, dysarthria, personality change, and pyramidal signs, may provide clinicians with a vital tool for improving diagnostic accuracy.
